# Cellular Pharmacokinetics and Intracellular Activity of Gepotidacin against Staphylococcus aureus Isolates with Different Resistance Phenotypes in Models of Cultured Phagocytic Cells

**DOI:** 10.1128/AAC.02245-17

**Published:** 2018-03-27

**Authors:** Frédéric Peyrusson, Paul M. Tulkens, Françoise Van Bambeke

**Affiliations:** a Pharmacologie cellulaire et moléculaire, Louvain Drug Research Institute, Université catholique de Louvain, Brussels, Belgium

**Keywords:** gepotidacin, MRSA, *Staphylococcus aureus*, daptomycin, linezolid, macrolides, monocytes, moxifloxacin, topoisomerases, vancomycin, THP-1 monocytes

## Abstract

Gepotidacin (GSK2140944), a novel triazaacenaphthylene bacterial topoisomerase inhibitor, is currently in clinical development for the treatment of bacterial infections. This study examined *in vitro* its activity against intracellular Staphylococcus aureus (involved in the persistent character of skin and skin structure infections) by use of a pharmacodynamic model and in relation to cellular pharmacokinetics in phagocytic cells. Compared to oxacillin, vancomycin, linezolid, daptomycin, azithromycin, and moxifloxacin, gepotidacin was (i) more potent intracellularly (the apparent bacteriostatic concentration [*C_s_*] was reached at an extracellular concentration about 0.7× its MIC and was not affected by mechanisms of resistance to the comparators) and (ii) caused a maximal reduction of the intracellular burden (maximum effect) of about −1.6 log_10_ CFU (which was better than that caused by linezolid, macrolides, and daptomycin and similar to that caused by moxifloxacin). After 24 h of incubation of infected cells with antibiotics at 100× their MIC, the intracellular persisting fraction was <0.1% with moxifloxacin, 0.5% with gepotidacin, and >1% with the other drugs. The accumulation and efflux of gepotidacin in phagocytes were very fast (*k*_in_ and *k*_out_, ∼0.3 min^−1^; the plateau was reached within 15 min) but modest (intracellular concentration-to-extracellular concentration ratio, ∼1.6). In cell fractionation studies, about 40 to 60% of the drug was recovered in the soluble fraction and ∼40% was associated with lysosomes in uninfected cells. In infected cells, about 20% of cell-associated gepotidacin was recovered in a sedimentable fraction that also contained bacteria. This study highlights the potential for further study of gepotidacin to fight infections where intracellular niches may play a determining role in bacterial persistence and relapses.

## INTRODUCTION

In spite of the availability of a large array of both old and newly approved antibiotics active against Gram-positive bacteria, Staphylococcus aureus is still considered by the World Health Organization (WHO) to be among the high-priority pathogens for which research and development of new therapies are needed (1). Antistaphylococcal antibiotics, indeed, should address the challenge not only of being active against multidrug-resistant strains that become increasingly prevalent but also of showing activity against latent forms of bacterial infection, which are often tolerant to antibiotic treatments. In this context, the capacity of S. aureus to survive within the host cells is considered to play a critical role in the persistence and/or recurrence of infections since intracellular bacteria are largely protected against antimicrobial treatments and host immune defenses (2–4). To act upon intracellular bacteria, antibiotics need to fulfill a series of pharmacokinetic and pharmacodynamic criteria, which globally reflect the intracellular bioavailability of the drug and its capacity to express activity toward bacteria under the specific conditions prevailing in the infected compartment (5). Exploring these properties is thus of high interest for antibiotics acting on new targets.

**FIG 1:**
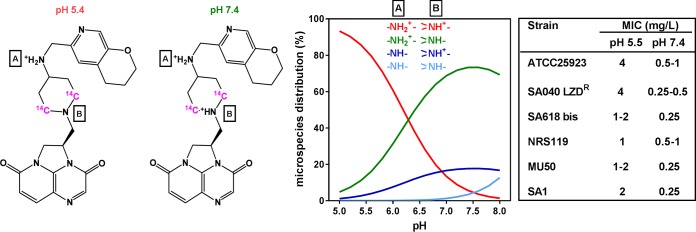
Structural formula and full IUPAC name of gepotidacin {(3*R*)-3-([4-((2*H*,3*H*,4*H*-pyrano[2,3-c]pyridin-6-ylmethyl)amino)piperidin-1-yl]methyl)-1,4,7- triazatricyclo[6.3.1.0^(4,12)]dodeca-6,8(12),9-triene-5,11-dione} (and the position of the ^14^C in the labeled compound) with the predominant ionized amino function(s) at pH 5.4 and at pH 7.4. The calculated pK_a_ values of these amino functions are 8.83 (A) and 6.20 (B). The calculated logD of the molecule is −0.73 at pH 8, −1.72 at pH 7, and −4.23 at pH 5. The graph shows the proportion of each ionized microspecies over the pH 5 to 8 range, with the protonation status of each of the two amino groups in these species being indicated. The table shows the MICs determined at pH 7.4 and 5.5 for the 6 strains under study. All physicochemical parameters were calculated using Reaxys software (Elsevier, 2016). LZD, linezolid.

Gepotidacin (originally known as GSK2140944; see its structure and ionization status at physiological pH in Fig. 1) is a novel antimicrobial agent belonging to the triazaacenaphthylene class of novel bacterial topoisomerase inhibitors, which are structurally different from fluoroquinolones and present a unique mechanism of action to impair DNA replication compared to fluoroquinolones. Instead of stabilizing DNA double-strand breaks like fluoroquinolones do, these antibiotics bind to a distinct site (6) and stabilize the precleavage type II topoisomerase enzyme-DNA complex prior to DNA cleavage, generating single-strand breaks (7, 8). Gepotidacin has a broad spectrum of activity, lower MICs, and more pronounced bactericidal effects against Gram-positive bacterial species than fluoroquinolones (9). It demonstrates robust activity against clinical isolates associated with skin and lower respiratory tract infections with MIC_90_s of ≤0.5 mg/liter, including against methicillin-resistant S. aureus (MRSA) and fluoroquinolone-resistant S. aureus or Streptococcus pneumoniae (10, 11). Gepotidacin has successfully completed a phase II clinical trial (12) in patients suffering from acute bacterial skin and skin structure infections (ABSSSIs) and also a study of its use for the treatment of uncomplicated urogenital infections caused by Neisseria gonorrhoeae.

The aims of the present study were (i) to measure the intracellular activity of gepotidacin using an *in vitro* pharmacodynamic model of intracellular infection in human THP-1 monocytes by various drug-susceptible and -resistant strains of S. aureus and to compare its activity with that of other antistaphylococcal agents and (ii) to examine in parallel the gepotidacin intracellular pharmacokinetics (influx/efflux, accumulation, and subcellular disposition) in human THP-1 monocytes and murine J774 macrophages. We found that gepotidacin is capable of reducing the intracellular bacterial burden to a larger extent than all comparator antibiotics except moxifloxacin, irrespective of the resistance phenotype of the strain. Gepotidacin showed a high rate of cellular uptake and efflux but low cellular accumulation levels at equilibrium, with most of the drug being localized in the soluble fraction and a smaller proportion being found in the phagolysosomal compartment where S. aureus sojourns.

## RESULTS

### Susceptibility of S. aureus strains to gepotidacin and comparator antibiotics.

Table 1 shows the MICs for gepotidacin and comparator antibiotics against all laboratory and clinical strains used in this study. These included strains harboring mechanisms of resistance to macrolides, linezolid, daptomycin, vancomycin, and fluoroquinolones (target mutations [strain SA618 bis] or efflux transporter NorA [strain SA1]). Gepotidacin consistently showed low MICs (0.25 to 1 mg/liter), whatever the resistance phenotype of the strain. Based on the physicochemical properties of gepotidacin (Fig. 1) that indicate a more hydrophilic character at acidic pH, MICs were also determined at pH 5.5 and found to be 3 to 4 log_2_ dilutions higher than those measured at neutral pH for most of the strains.

**TABLE 1:** Strains used in the study, strain origin, and MIC in broth^*a*^

Strain	Origin	MIC (mg/liter)									
		GEP	AZM	CLR	OXA	VAN	LZD	DAP	MXF	CIP	GEN
ATCC 25923	Laboratory^*b*^	0.5–1	1	0.25	0.25	1	2–4	1	0.03–0.0625	0.125–0.25	0.5
SA040 LZD^r^	*In vitro* mutant from clinical isolate^*c*^	0.25–0.5	2	0.25	0.25	1–2	**16**	**2**	0.125	ND	0.5
SA618 bis	Clinical^*d*^	0.25	ND	ND	**256**	**4**	2	**32**	**4**	ND	0.125
NRS119	Clinical^*e*^	0.5–1	**4**	2	**>256**	1	**128**	**2**	**4**	ND	**64**
MU50	Clinical^*f*^	0.25	**>256**	**>256**	**>256**	**8**	1	**8**	**4**	ND	**64**
SA1	Laboratory^*g*^	0.25	ND	ND	ND	ND	ND	ND	0.0625	**4**	ND

a Data in bold indicate values greater than the EUCAST resistant (r) clinical breakpoint values (European Committee on Antimicrobial Susceptibility Testing, 2017). Abbreviations: GEP, gepotidacin; AZM, azithromycin; CLR, clarithromycin; OXA, oxacillin; DAP, daptomycin; VAN, vancomycin; LZD, linezolid; MXF, moxifloxacin; CIP, ciprofloxacin; GEN, gentamicin; ND, not determined.

b Laboratory standard (ATCC, Manassas, VA).

c From P. Appelbaum, Hershey Medical Center, Hershey, PA (41). Selected by *in vitro* exposure of a clinical isolate to increasing concentrations of linezolid; unknown resistance mechanism.

d Respiratory tract infection. From P. Appelbaum, Hershey Medical Center, Hershey, PA (42). Described as a MRSA and heterogeneous vancomycin-intermediate S. aureus strain.

e Peritonitis (43). Described as a MRSA and linezolid-resistant strain with a mutated domain V in 23S RNA.

f ATCC 700699 (ATCC, Manassas, VA). Surgical wound infection, vancomycin-intermediate S. aureus (44).

g *In vitro* mutant overexpressing NorA; selected by *in vitro* exposure of ATCC 25923 to increasing concentrations of ethidium bromide (45); from Claudine Quentin, Université de Bordeaux 2, Bordeaux, France.

### Cellular viability.

We first checked for the lack of cytotoxicity of gepotidacin toward eukaryotic cells by measuring the release of the cytosolic enzyme lactate dehydrogenase (LDH) from THP-1 monocytes under the conditions used for further experiments. This release was lower than 5% and not significantly different from control values after 24 h of incubation with gepotidacin at concentrations up to 50 mg/liter (50 times the highest MIC against S. aureus in the present study).

### Extracellular and intracellular activity of gepotidacin and comparators. (i) Extracellular activity (cation-adjusted Mueller-Hinton broth [CA-MHB]).

**FIG 2:**
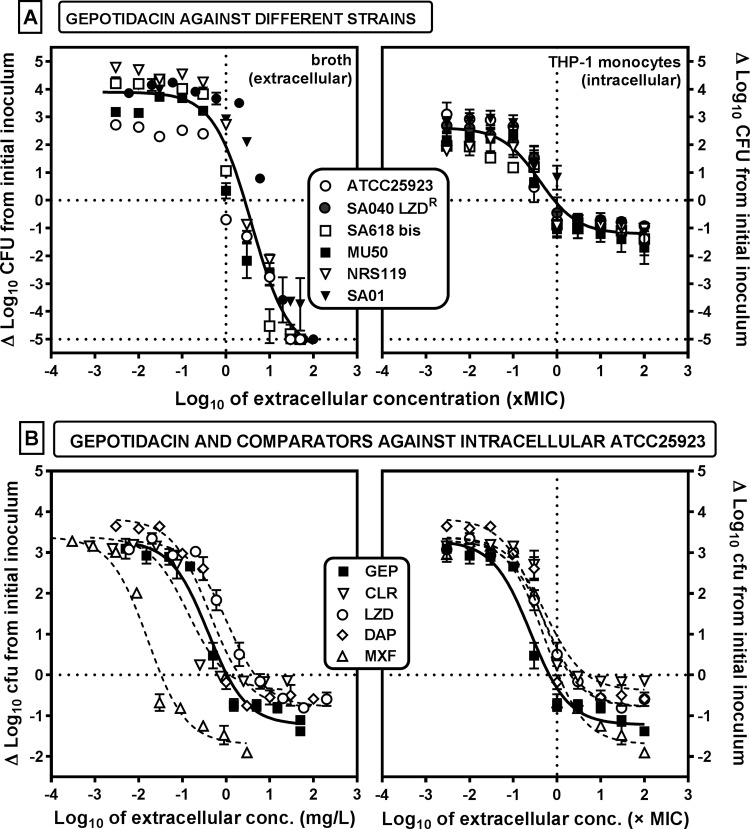
(A) Concentration-response curves of gepotidacin against extracellular (left) and intracellular (right) forms of S. aureus strains with different resistance phenotypes (Table 1). The graphs show the changes in the number of CFU from the initial inoculum per milliliter of broth (left) or per milligram of cell protein in THP-1 monocytes (right) after 24 h of incubation with increasing extracellular concentrations (expressed in multiples of the MIC). (B) Concentration-response curves of the intracellular activity of gepotidacin (GEP) and comparators (clarithromycin [CLR], linezolid [LZD], daptomycin [DAP], moxifloxacin [MXF]) against strain ATCC 25923 (methicillin-susceptible S. aureus). The graphs show the changes in the number of CFU from the initial inoculum per milligram of cell protein in THP-1 cells after 24 h of incubation with increasing extracellular concentrations expressed either as the total extracellular concentration in milligrams per liter (left) or in multiples of the corresponding MIC (right). The horizontal dotted lines highlight a static effect (*C_s_*) and the lowest limit of detection (a decrease in the number of CFU of 5 log_10_ units compared to the initial inoculum; panel A only), and the vertical dotted lines highlight the MIC, when applicable. All data are means ± SEMs (*n* = 3).

The extracellular activity of gepotidacin against 6 strains of S. aureus that included 5 strains resistant to at least one comparator antibiotic was evaluated. To this effect, the residual numbers of CFU were measured after 24 h of incubation with extracellular concentrations ranging from 0.001× to 100× the MIC of each strain (Fig. 2A, left) in order to obtain full concentration-effect curves (see Table 2 for pharmacological descriptors). Gepotidacin showed a bacteriostatic effect at a concentration close to its MIC and a bactericidal effect (3-log_10_-CFU decrease) at 10× its MIC for all strains. A single sigmoid function could be satisfactorily fitted to the whole data set when the change in the number of CFU was expressed as a function of equipotent concentrations (multiples of the MIC), demonstrating that gepotidacin activity was independent of the phenotype of the resistance of the strains to the comparators.

**TABLE 2:** Pharmacological parameters and statistical analysis of the dose-response curves of antibiotics against all strains tested in THP-1 monocytes

Antibiotic and strain	*E*_min_^*a*^^,^^*d*^	*E*_max_^*b*^^,^^*d*^	*C_s_*^*c*^^,^^*d*^		*R*^2^
			mg/liter	Multiple of MIC	
Gepotidacin					
Pooled data^*e*^					
Extracellular	3.9 (3.46 to 4.38)	−5.5 (−6.4 to −4.6)	Not applicable	2.69 (2.42 to 2.98)	0.91
Intracellular	2.62 (2.46 to 2.77)	−1.21 (−1.35 to −1.07)	Not applicable	0.68 (0.51 to 0.84)	0.9
Individual strains^*f*^					
ATCC 25923	3.29 (2.92 to 3.66) A	−1.22 (−1.49 to −0.96) AB, a	0.34 (0.26 to 0.42) A, ab	0.68 (0.51 to 0,84) A, a	0.95
SA040 LZD^r^	2.73 (2.45 to 3.02) AB	−0.99 (−1.26 to −0.72) A, a	0.28 (0.18 to 0.37) A, ab	0.55 (0.37 to 0.72) A, a	0.95
SA618 bis	1.94 (1.61 to 2.27) C	−1.28 (−1.59 to −0.97) A, a	0.16 (0.15 to 0.16) A, b	0.63 (0.61 to 0.65) A, a	0.9
NRS119	2.21 (1.89 to 2.53) BC	−1.15 (−1.43 to −0.87) AB, a	0.42 (0.34 to 0.51) A, a	0.84 (0.67 to 1.01) A, a	0.93
MU50	2.51 (2.15 to 2.87) C	−1.60 (−1.92 to −1.26) AB, a	0.16 (0.13 to 0.20) A, b	0.65 (0.52 to 0.79) A, a	0.94
SA1	2.99 (2.76 to 3.22) A	−1,07 (−1.32 to −0.82) A, a	0.46 (0.31 to 0.6) A, a	1.83 (1.23 to 2.42) A, b	0.96
Linezolid					
ATCC 25923	3.29 (3.10 to 3.49) A	−0.78 (−0.99 to −0.57) BC, a	4.60 (4.40 to 4.79) C, a	2.30 (2.20 to 2.40) A, a	0.98
SA040 LZD^r^	3.14 (2.87 to 3.40) AB	−0.25 (−0.51 to 0.01) B, a	58.6 (27.8 to 108) B, b	3.66 (1.74 to 6.79) B, a	0.96
NRS119	2.69 (2.45 to 2.93) BC	−1.31 (−1.82 to −0.81) AB, a	69.3 (47.1 to 96) B, b	1.08 (0.74 to 1.50) AB, a	0.95
Clarithromycin					
ATCC 25923	3.35 (3.06 to 3.65) A	−0.38 (−0.73 to −0.04) C, a	1.23 (1.10 to 1.34) AB	4.90 (4.42 to 5.37) B	0.95
MU50	2.05 (1.93 to 2.17) C	1.98 (1.89 to 2.07) C, b	No convergence	No convergence	0.08
Daptomycin					
ATCC 25923	3.84 (3.42 to 4.25) A	−0.79 (−1.19 to −0.40) BC, a	2.35 (1.03 to 3.67) B, b	2.35 (1.03 to 3.67) A, a	0.96
SA040 LZD^r^	3.25 (2.91 to 3.59) AB	−0.35 (−0.62 to −0.08) B, a	2.36 (1.88 to 2.91) A, b	1.18 (0.95 to 1.94) AB, a	0.95
SA618bis	1.99 (1.70 to 2.29) C	−0.76 (−1.08 to −0.42) A, a	15.7 (14.90 to 16.50) C, a	0.49 (0.47 to 0.52) A, a	0.93
NRS119	2.44 (2.20 to 2.68) BC	−0.44 (−0.67 to −0.20) B, a	3.85 (3.61 to 4.10) A, b	1.92 (1.80 to 2.05) B, a	0.95
MU50	2.07 (1.87 to 2.27) C	−0.70 (−0.93 to −0.47) B, a	16.3 (15.20 to 18) C, a	2.04 (1.90 to 2.23) B, a	0.96
Moxifloxacin					
ATCC 25923	3.39 (3.02 to 3.76) A	−1.70 (−2.11 to −1.30) A, ab	0.03 (0.03 to 0.04) A, c	1.10 (0.95 to 1.25) A, b	0.97
SA040 LZD^r^	2.58 (2.29 to 2.88) AB	−1.14 (−1.46 to −0.81) A, a	0.54 (0.52 to 0.56) A, bc	4.33 (4.17 to 4.49) B, a	0.96
SA618bis	2.49 (2.20 to 2.79) C	−1.23 (−1.61 to −0.84) A, a	7.10 (4.51 to 9.68) B, a	1.77 (1.13 to 2.42) B, b	0.94
NRS119	2.23 (1.91 to 2.54) BC	−2.11 (−2.54 to −1.68) A, ab	4.50 (3.12 to 5.88) A, ab	1.12 (0.78 to 1.47) AB, b	0.95
MU50	2.13 (1.82 to 2.43) C	−2.42 (−2.91 to −1.93) A, b	3.81 (3.75 to 3.88) B, abc	0.95 (0.94 to 0.97) B, b	0.95
SA1	2.97 (2.66 to 3.29) A	−1.29 (−1.62 to −0.96) A, a	0.10 (0.08 to 0.11) A, c	1.52 (1.30 to 1.74) A, b	0.97
Ciprofloxacin					
SA1	3.13 (2.67 to 3.58) A	−1.25 (−1.64 to −0.85) A	3.36 (2.97 to 3.76) B	0.84 (0.74 to 0.94) A	0.94

a Increase in the number of CFU (in log_10_ units, with the confidence interval being given in parentheses) at 24 h from the corresponding initial inoculum, as extrapolated from the Hill equation of the concentration-effect response for an infinitely low antibiotic concentration.

b Decrease in the number of CFU (in log_10_ units, with the confidence interval being given in parentheses) at 24 h from the corresponding initial inoculum, as extrapolated from the Hill equation of the concentration-effect response for an infinitely large antibiotic concentration.

c Extracellular antibiotic concentration (with the confidence interval being given in parentheses) resulting in no apparent bacterial growth, as calculated from the Hill equation of the concentration-response curve.

d Statistical analyses were performed by one-way analysis of variance with the Tukey-Kramer multiple-comparison *t* test. For *E*_max_ and *C_s_*, values followed by different uppercase letters (response of antibiotics to the same strain) or lowercase letters (response of the strains to the same antibiotic) are significantly different from each other (*P* < 0.05). For *E*_min_, values followed by different uppercase letters (response of strains disregarding the antibiotic used [since its concentration is infinitely low]) are significantly different from each other (*P* < 0.05).

e One regression for all strains (Fig. 2A).

f See Fig. 2B and 3.

### (ii) Intracellular activity (THP-1 human monocytes).

The same type of experiment was then performed to evaluate the activity of gepotidacin against bacteria phagocytized by THP-1 monocytes (see Fig. 2A, right, and Table 2 for the values of the pharmacological descriptors). Gepotidacin activity developed in a concentration-dependent fashion with similar pharmacodynamic parameters for all strains, whatever their phenotype of resistance to the other antibiotics. However, the maximal relative efficacy (maximum effect [*E*_max_], which is the decrease in the number of CFU compared to the original inoculum for an infinitely large extracellular antibiotic concentration) against intracellular bacteria was considerably lower (less negative) than that against extracellular bacteria, with a value of only about −1.2 log_10_ CFU compared to the postphagocytosis bacterial burden. In contrast, the apparent bacteriostatic concentration (*C_s_*; the extracellular concentration of drug [expressed in milligrams per liter or in multiples of the MIC] causing no apparent change in the number of CFU) remained close to the MIC, as for the extracellular bacteria. In a next step, the intracellular activity of gepotidacin was compared with that of other antibiotics toward the fully susceptible S. aureus strain ATCC 25923. Data are shown in Fig. 2B, with extracellular concentrations being expressed in milligrams per liter (left) or as a multiple of the MIC (right), and the corresponding pharmacodynamic parameters are presented in Table 2. Moxifloxacin was the most potent (lowest *C_s_*) among the drugs tested owing its low MIC value, but it was also the most effective, with an *E*_max_ at −1.7 log_10_ CFU. The other drugs were as potent as or less potent than gepotidacin but also less effective (i.e., they had a less negative *E*_max_) than gepotidacin. When the antibiotics were compared at equipotent concentrations, their *C_s_* values were close to (0.7 to 5 times) their respective MICs.

We then compared the intracellular activity of gepotidacin and other antibiotics against strains harboring resistance mechanisms (see Fig. 3 for a direct comparison of gepotidacin and antibiotics affected by resistance and Table 2 for numerical data, including data for additional susceptible and resistant strains tested with each antibiotic). In all cases, the intracellular *C_s_* remained close to the MIC and was therefore shifted to much larger concentrations for antibiotics affected by resistance but not for gepotidacin. The maximal relative efficacy of each antibiotic (*E*_max_) was unchanged, except for clarithromycin against the MU50 strain, for which a Hill function could not be fitted to the data as we could not expose it to concentrations exceeding its MIC (>256 mg/liter).

**FIG 3:**
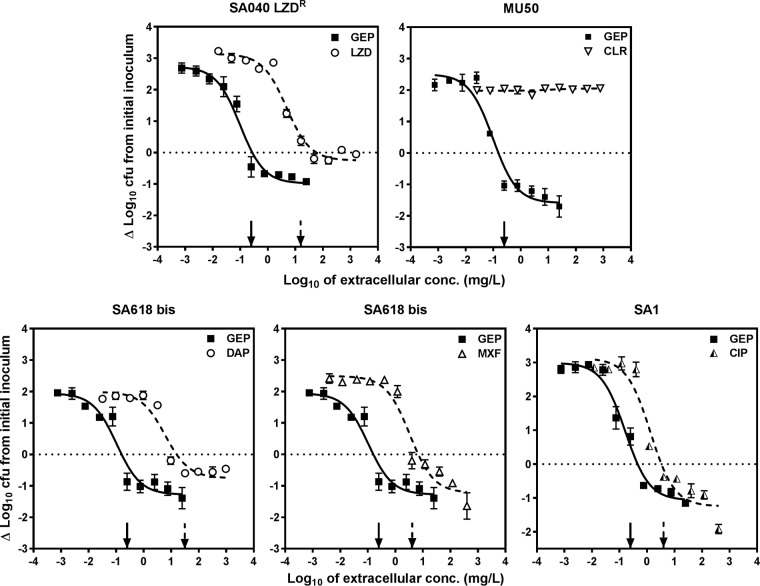
Intracellular concentration-response curves of gepotidacin (GEP) against strains with resistance to the comparators (clarithromycin [CLR], linezolid [LZD], daptomycin [DAP], moxifloxacin [MXF], ciprofloxacin [CIP]) and phagocytized by THP-1 monocytes (only data pertaining to gepotidacin and the key comparators for each strain are shown on the graphs; see Table 2 for the pharmacological descriptors of the activity of the other drugs). The ordinate shows the changes in the log_10_ number of CFU per milligram of cell protein after 24 h of incubation compared to the initial inoculum. The abscissa shows the drug concentration, expressed as the log_10_ total extracellular concentrations in milligrams per liter. For all panels, the plain and dotted arrows point to the MICs of gepotidacin and its comparators, respectively, and the horizontal dotted line shows a static effect (*C_s_*). Data are means ± SEMs (*n* = 3).

**FIG 4:**
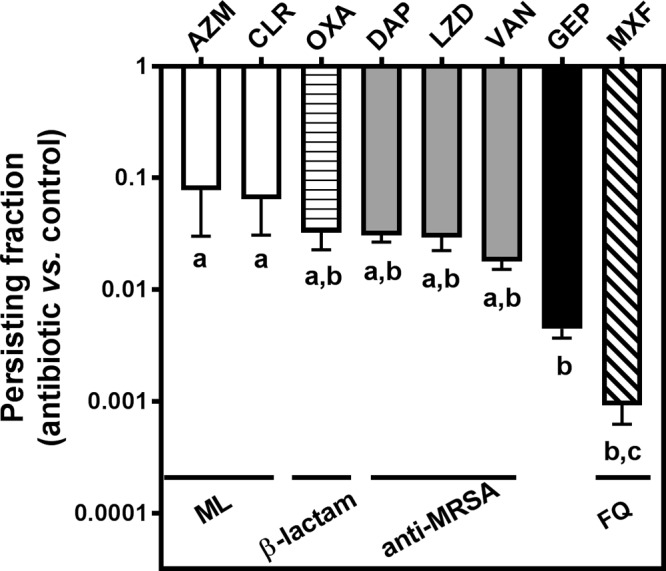
Evaluation of the intracellular persisting fraction. For each tested antibiotic, the bar shows the ratio between the log_10_ number of CFU per milligram of protein after 24 h of incubation with 100× MIC of antibiotic (or the highest value tested if the MIC was >256 mg/liter) and the log_10_ number of CFU per milligram of protein under control conditions (24 h of incubation in the presence of gentamicin at its MIC to avoid extracellular contamination [15]). Data are means ± SEMs of the values calculated for the 6 strains investigated in three independent experiments. AZM, azithromycin; CLR, clarithromycin; OXA, oxacillin; DAP, daptomycin; LZD, linezolid; VAN, vancomycin; GEP, gepotidacin; MXF, moxifloxacin; ML, macrolide; FQ, fluoroquinolone. Statistical analysis was performed by one-way analysis of variance with Tukey's *post hoc* test. Data sets with different letters are significantly different from one another (*P* < 0.05).

Lastly, we compared the persisting bacterial fraction in cells infected by each of the investigated strains and exposed for 24 h to high concentrations of each of the drugs under study (Fig. 4). The highest persisting bacterial fraction (>1%) was observed for infected cells exposed to macrolides, oxacillin (β-lactam), or each of the 3 anti-MRSA antibiotics (daptomycin, linezolid, and vancomycin, from highest to lowest), and the lowest one (<0.1%) was observed after incubation with moxifloxacin. With gepotidacin, the persisting bacterial fraction (0.5%) was slightly higher than that observed with moxifloxacin but significantly lower than that observed with the other drugs.

### Cellular influx, accumulation, and efflux of gepotidacin.

**FIG 5:**
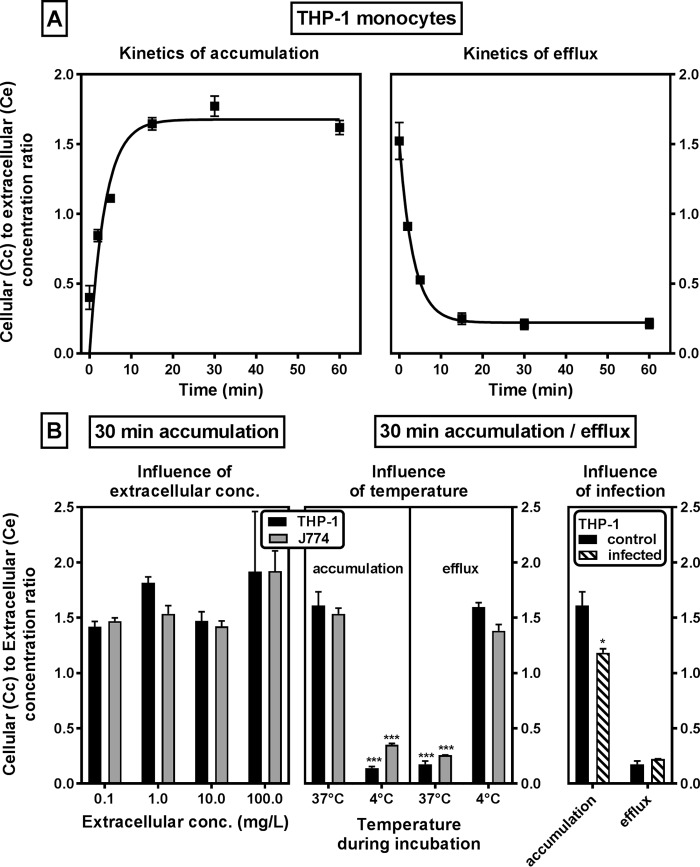
Accumulation and release of gepotidacin in human THP-1 monocytes and murine J774 macrophages. (A) THP-1 monocytes were incubated with [^14^C]gepotidacin at a fixed concentration (1 mg/liter) and collected (accumulation) (left) or incubated for 30 min and then returned to drug-free medium (efflux) (right). (B) (Left) THP-1 and J774 cells were incubated with [^14^C]gepotidacin at different extracellular concentrations for 30 min; (middle) both cell lines were incubated for 30 min with 1 mg/liter [^14^C]gepotidacin and collected (accumulation) or returned to drug-free medium for 30 min at the temperatures indicated (efflux); (right) same experiment as in the middle panel, but uninfected and infected THP-1 cells were compared at 37°C. For each graph, the ordinate shows the apparent ratio between the cellular and the extracellular concentrations. Data are means ± SDs (*n* = 3). Statistical analysis was performed by analysis of variance with the Tukey-Kramer multiple-comparison *t* test (B, left) and an unpaired multiple *t* test (B, middle and right).

The kinetics of accumulation and efflux as well as the level of accumulation of gepotidacin at equilibrium were then determined in uninfected THP-1 monocytes and mouse J774 macrophages exposed to a microbiologically meaningful and clinically achievable (12) extracellular concentration of 1 mg/liter (Fig. 5A). Gepotidacin uptake proceeded according to a one-phase exponential association at a constant rate of 0.27 min^−1^, to reach an apparent stable cellular concentration 1.7-fold higher than the extracellular one after 15 min. Efflux occurred at the same rate (constant, 0.30 min^−1^) and was almost complete (residual apparent accumulation, 0.2) after approximately 15 min. The accumulation level at equilibrium (after 30 min) was similar in THP-1 and J774 cells and not influenced by the extracellular concentration over a broad range (0.1 to 100 mg/liter; Fig. 5B, left). In both cell types, accumulation was markedly reduced when cells were incubated at 4°C instead of 37°C. Conversely, efflux was completely abolished when cells loaded at 37°C were reincubated at 4°C in an antibiotic-free medium (Fig. 5B, middle). Lastly, the accumulation of gepotidacin in infected versus noninfected THP-1 cells incubated for 30 min with 1 mg/liter gepotidacin was measured, and no major difference was observed (Fig. 5B, right). Likewise, residual accumulation was similar under both conditions after 30 min reincubation in gepotidacin-free medium at 37°C.

### Subcellular distribution of gepotidacin in uninfected and infected cells.

**FIG 6:**
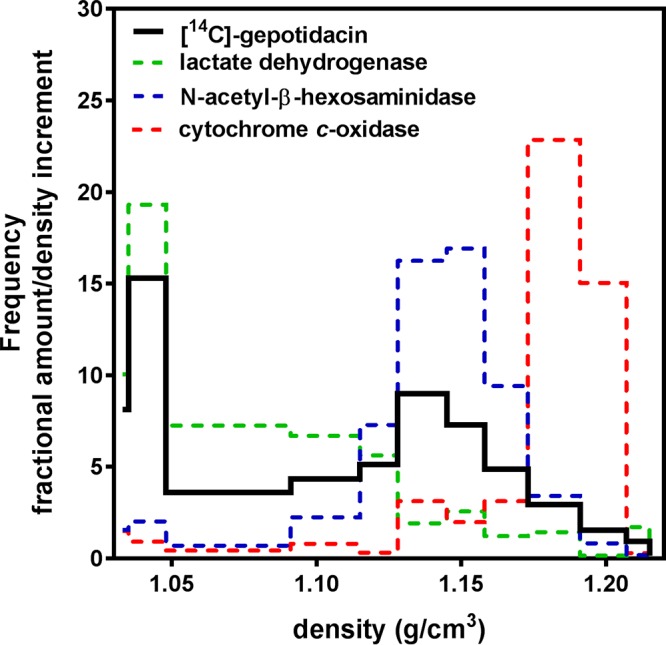
Fractionation of cytoplasmic extracts of J774 macrophages by isopycnic centrifugation in a linear sucrose gradient (collected in 12 discrete fractions). Cells were incubated with 1 mg/liter [^14^C]gepotidacin for 30 min prior to collection. Results are presented as histograms of the density distribution of [^14^C]gepotidacin and of the marker enzymes (lactate dehydrogenase, cytosol; cytochrome *c*-oxidase, mitochondria; *N*-acetyl-β-hexosaminidase, lysosomes). The abscissa is the density span of the gradient. The ordinate is the frequency of the distribution, defined as the fractional amount of activity recovered in each fraction divided by the density interval of that fraction. The surface of each section of the diagrams therefore represents the fraction of each constituent recovered in the corresponding density span. Data are from a single experiment that was repeated with very similar results.

**FIG 7:**
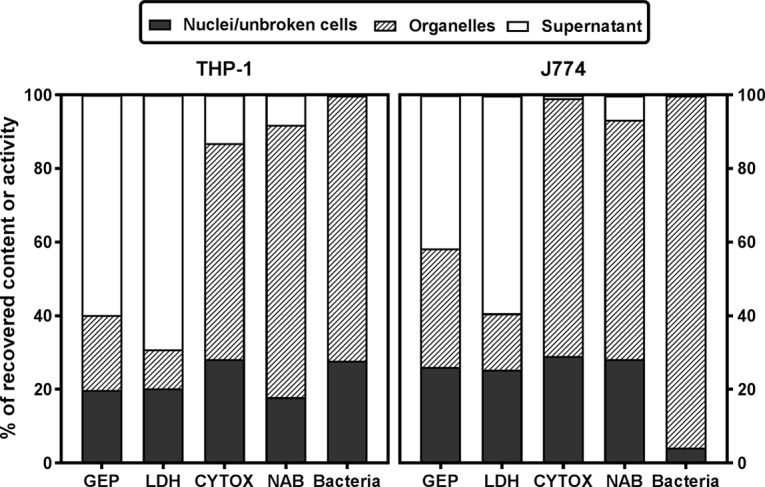
Subcellular distribution of [^14^C]gepotidacin, marker enzymes (lactate dehydrogenase [LDH], cytosol; cytochrome *c*-oxidase [CYTOX], mitochondria; *N*-acetyl-β-hexosaminidase [NAB], lysosomes) and bacteria in homogenates of THP-1 (left) or J774 (right) cells, expressed as a percentage of the total recovered amount or activity. Cells were infected for 1 h and returned to fresh medium for 2 h to allow complete internalization of the bacteria and exposed to 0.1 mg/liter of [^14^C]gepotidacin for 30 min prior to collection. Homogenates were separated into 3 fractions by centrifugation at increasing centrifugal fields (for the main cytological content, i.e., nuclei and unbroken cells, organelles [mitochondria, lysosomes], and the final supernatant). Data are the means from 2 experiments with similar results.

The subcellular distribution of gepotidacin was studied in J774 macrophages incubated for 30 min with 1 mg/liter gepotidacin. Figure 6 shows the distribution of the radiotracer and of markers of the cytosol (lactate dehydrogenase), lysosomes (*N*-acetyl-β-hexosaminidase), and mitochondria (cytochrome *c*-oxidase) in a sucrose gradient after isopycnic centrifugation of the cell homogenate. Enzymatic markers were distributed in different fractions of the gradient, with lactate dehydrogenase being located mainly in the lighter fractions, cytochrome *c*-oxidase being located in the heavier fractions, and *N*-acetyl-β-hexosaminidase being located in the fraction with a density of about 1.13. Gepotidacin showed a bimodal distribution, with 46% of the radioactivity showing a distribution similar to that of lactate dehydrogenase and 45% showing a distribution similar to that of *N*-acetyl-β-hexosaminidase. This experiment could not be performed with THP-1 cells, since lysosomal and mitochondrial markers equilibrate in the same fractions for these cells. We therefore also studied the distribution of gepotidacin in parallel in J774 and THP-1 homogenates that had been more grossly fractionated by differential centrifugation in order to separate organelles on the basis of their size (Fig. 7). These experiments were performed in infected cells incubated for 2 h at 37°C after phagocytosis of bacteria to allow for their complete internalization and then for 30 min with 0.1 mg/liter of gepotidacin (sub-MIC, to maintain bacterial viability). In both cell types, [^14^C]gepotidacin was mainly recovered in the final supernatant (60% and 40% in THP-1 and J774 cells, respectively), with smaller amounts being found in the organelle-containing fraction (20% and 30% in THP-1 and J774 cells, respectively) and the remainder being found in the nucleus/unbroken cell fraction. As previously described, lactate dehydrogenase was mostly recovered in the soluble fraction, and cytochrome *c*-oxidase and *N*-acetyl-β-hexosaminidase were mostly recovered in the organelle fraction, together with bacteria (13). Cell fractionation experiments performed with uninfected THP-1 and J774 cells according to the same protocol showed a similar distribution of the drug and of the marker enzymes (data not shown).

## DISCUSSION

This study is, to the best of our knowledge, the first one to document the cellular pharmacokinetics and intracellular activity of this new triazaacenaphthylene bacterial topoisomerase inhibitor. Because its mechanism of action is distinct from that of currently approved antibiotics, it is not surprising that gepotidacin shows the same level of activity against fully susceptible S. aureus strains and against strains resistant to the comparators used in this study as well as to other antistaphylococcal drugs (10, 11). For fluoroquinolones, we show here that gepotidacin activity is maintained not only against strains harboring mutations in fluoroquinolone targets but also against those expressing the NorA efflux pump, which affects ciprofloxacin and other hydrophilic fluoroquinolones (14).

Considering the results of our intracellular pharmacodynamic experiments as a whole, we can apply to gepotidacin the general concepts previously described and discussed at length for other classes of bactericidal antistaphylococcal antibiotics in our previous publications (15, 16), namely, that most of them show activity against the intracellular forms of S. aureus but (i) that their maximal relative efficacy (*E*_max_) is markedly reduced compared to what is observed against extracellular bacteria (17), while (ii) the extracellular concentration needed to obtain an intracellular static effect (*C_s_*) remains close to the MIC, as determined in broth, denoting no loss of relative potency. Globally, however, gepotidacin stands as one of the most effective drugs tested in this model so far (see reference 5 for a review) after the lipoglycopeptide oritavancin (18) and the fluoroquinolones moxifloxacin and delafloxacin with activity against Gram-positive bacteria (19). The lower persisting fraction observed here for bacteria exposed to gepotidacin and moxifloxacin than for bacteria exposed to the comparators illustrates the potential interest of targeting topoisomerase enzymes when dealing with intracellular forms of infections by S. aureus. Assessing the maximal intracellular relative activity (*E*_max_) of antibiotics and measuring the bacterial persisting fraction are probably of high clinical relevance, as we know that even low inocula of intracellular S. aureus are capable of causing severe infections *in vivo* (20) and that intracellular survival is associated with the recurrent character of several staphylococcal infections in humans (4, 21, 22). Focusing on gepotidacin, our study shows that this antibiotic consistently decreases the intracellular bacterial burden to a value close to the maximal relative efficacy (*E*_max_) when present in the extracellular medium at concentrations that can be reached in human serum, based on the results of the recently published clinical trial (maximum concentration [*C*_max_] range, 2.4 to 8.8 mg/liter, depending on the dose and administration route [12]; no minimum concentration values were available).

A striking observation, however, is that the intracellular activity of gepotidacin develops without marked accumulation in cells (apparent cellular concentration-to-extracellular concentration ratio, approximately 1.6), in sharp contrast to fluoroquinolones, which accumulate 5 to 20 times (23), or macrolides, which accumulate to even much higher values (24). This is actually in accordance with a number of previous observations made with our model, where we showed that there is no direct correlation between the global cellular accumulation of antibiotics and the level of their intracellular activity when comparing drugs of different pharmacological classes (see references 13, 15, 23, and 25 for typical examples). Yet, the molecular reasons for a lack of such a correlation remain to be established for most antibiotics. Of note, however, static effects are obtained intracellularly for extracellular concentrations close to the MIC for both gepotidacin and moxifloxacin. This would tend to suggest a higher intracellular bioavailability of gepotidacin than of moxifloxacin and/or a defeating effect exerted by the intracellular milieu on the potency of moxifloxacin. Of note also, both gepotidacin and moxifloxacin showed lower intrinsic activity (higher MIC) at acidic pH, which may contribute to a reduction of their intracellular potency against S. aureus, which thrives in acidic intracellular compartments, counteracting the beneficial effect of their accumulation.

Turning our attention to the cellular pharmacokinetics of gepotidacin, our data suggest that it enters eukaryotic cells by passive diffusion since (i) the rates of influx and efflux are high and very similar to each other, with no evidence of saturation over the range of concentrations investigated (including concentrations up to 12-fold higher than the human *C*_max_ [12] and 100 times the MIC), and (ii) accumulation was almost completely impaired at 4°C (a condition which considerably reduces membrane fluidity). The intracellular disposition of gepotidacin (partly in lysosomes and partly in the cytosol) is also consistent with its character as a weak base, with pK_a_s being close to the range of pHs between lysosomes (about pH 5) and the cytosol (about pH 7), as previously observed for other antibiotics (see reference 26 for models and references 24 and 27 for typical examples). Of interest, this also means that part of the accumulated drug is located in the same compartment as bacteria (see the results of fractionation studies with infected cells as well as data demonstrating a phagolysosomal localization for S. aureus in phagocytic cells [15, 28, 29]). While this part seems only minor (about 20%), we cannot exclude the possibility of a redistribution of the drug from lysosomes to the cell supernatant during the homogenization and fractionation processes, which are associated with an extensive dilution of the cellular material.

Gepotidacin has now successfully completed a phase II clinical trial for the treatment of acute bacterial skin and skin structure infections suspected or confirmed to be caused by Gram-positive bacteria (12). Pharmacodynamic studies in a model of murine lung infection have demonstrated the area under the concentration-time curve-dependent activity and a pharmacokinetic profile that supports further investigation of this compound for the treatment of infections caused by MRSA (30). The present study adds that gepotidacin may be of particular interest as an agent that acts upon intracellular S. aureus, which may play a critical role in persistent or recurrent skin or respiratory tract infections (21, 31, 32).

## MATERIALS AND METHODS

### Antibiotics and main products.

Gepotidacin (Fig. 1) and [^14^C]gepotidacin (specific activity, 59.9 mCi/mmol; radiochemical purity, 99.7%) were obtained from GlaxoSmithKline plc (Collegeville, PA). Stock solutions of unlabeled gepotidacin were prepared in dimethyl sulfoxide at a concentration of 50 mg/liter and thereafter diluted in water to the desired concentration. The radiolabeled compound was added in a tracing amount to stock solutions of unlabeled gepotidacin in order to obtain appropriate signals under our experimental conditions. The stability of gepotidacin under our experimental conditions was checked by determining the MICs of culture medium (cation-adjusted Mueller-Hinton broth [CA-MHB; Becton, Dickinson and Company, Franklin Lakes, NJ], RPMI 1640 supplemented or not supplemented with 10% fetal bovine serum [FBS]) spiked with gepotidacin that had been preincubated or not preincubated for 24 h at 37°C and geometrically diluted in a microtiter plate inoculated with S. aureus SA618 bis. No difference was seen in the concentration of gepotidacin needed to inhibit bacterial growth for samples preincubated for 24 h or not, demonstrating its stability under our conditions. The following antibiotics were obtained as microbiological standards: azithromycin and clarithromycin, from SMB-Galephar (Marche-en-Famenne, Belgium); moxifloxacin HCl, from Bayer AG (Wuppertal, Germany); and oxacillin monohydrate and gentamicin sulfate, from Sigma-Aldrich (St. Louis, MO). The other antibiotics were obtained as the corresponding branded products that are registered for human parenteral use in Belgium and that comply with the provisions of the European Pharmacopoeia (vancomycin as Vancomycine Mylan [Mylan Inc., Canonsburg, PA] and linezolid as Zyvoxid [Pfizer Inc., New York, NY]). Human serum was obtained from Biowest SAS (Nuaillé, France), and cell culture media and sera were obtained from Gibco/Life Technologies Corporation (Paisley, United Kingdom). Unless stated otherwise, all other products were obtained from Sigma-Aldrich or Merck KGaA (Darmstadt, Germany).

### Cell lines.

Experiments were performed using (i) human THP-1 cells (a myelomonocytic cell line [33]), purchased as clone ATCC TIB-202 from the American Type Culture Collection, Manassas, VA, and (ii) murine J774 macrophages (derived from a reticulosarcoma [34]), originally obtained from Sandoz Forschung Laboratories, Vienna, Austria. Both cell lines were maintained in our laboratory as previously described in RPMI 1640 medium supplemented with 10% fetal bovine serum in a 5% CO_2_ atmosphere (35, 36).

### Bacterial strains and susceptibility testing.

The laboratory and clinical strains used in the present study are listed in Table 1 with information on their origins and resistance phenotypes. MICs were determined by microdilution in CA-MHB following the recommendations of the Clinical and Laboratory Standards Institute (37). In specific experiments, the medium was adjusted to pH 5.5 using HCl.

### Assessment of viability of THP-1 monocytes.

Cell viability in the presence of increasing concentrations of gepotidacin was evaluated by measuring the release of the cytosolic enzyme lactate dehydrogenase (LDH) in the culture medium after 24 h of incubation using a Cytotoxicity Detection Kit^Plus^ (LDH) (Roche Diagnostics GmbH, Manheim, Germany) following the manufacturer's instructions. The release of LDH was expressed as the percentage of activity detected in the medium compared to the total enzymatic activity in the culture.

### Determination of extracellular and intracellular activities of antibiotics.

For extracellular activity, experiments were performed in CA-MHB with an initial inoculum of 10^6^ CFU/ml. Bacteria were incubated with antibiotics over a broad range of extracellular concentrations for 24 h, after which aliquots were taken, appropriately diluted, and plated on agar. Results are expressed as the change in the number of CFU per milliliter from the initial inoculum, as assessed by colony counting. Bactericidal activity was defined as a reduction of 99.9% (a 3-log_10_-CFU/ml decrease) of the total counts. For intracellular activity, cell infection was performed as described previously (15). In brief, bacteria were opsonized with human serum (10% in RPMI 1640) for 30 min at 37°C. Bacteria were then incubated at an inoculum of 4 bacteria per cell for 1 h to allow phagocytosis. After removal of the medium and washing of the cells with phosphate-buffered saline (PBS), infected cells were incubated for 45 min with gentamicin at 100× its MIC to eliminate extracellular bacteria, washed with PBS to eliminate gentamicin, and reincubated for 24 h with increasing concentrations of antibiotics. The cells were then washed with PBS and collected in H_2_O. The numbers of CFU were determined by plating, and proteins were assayed by the Folin-Ciocalteu method. Results were expressed as the change in the number of CFU per milligram of cell protein from the postphagocytosis inoculum. Data from concentration-response experiments were used to fit a sigmoidal function (Hill equation) and calculate pertinent pharmacodynamic parameters, i.e., *E*_max_ (maximal relative efficacy, which is the decrease in the number of CFU compared with the original inoculum for an infinitely large extracellular antibiotic concentration [note that this parameter is negative if killing occurs and is more negative for drugs with greater efficacy]), the minimum effect (*E*_min_; minimal relative efficacy, which is the increase in the number of CFU compared with the original inoculum for an infinitely low extracellular antibiotic concentration [this parameter essentially describes bacterial growth]), and *C_s_* (see references 13 and 17 for further details). The Hill equation was also used to calculate the intracellular persisting bacterial fraction, which was defined as the ratio between the residual number of CFU per milligram of protein after 24 h exposure to 100× MIC of the antibiotic (or the maximal reachable concentration for isolates against which the MIC of the corresponding antibiotic was >128 mg/liter) and after 24 h of incubation in the absence of the added antibiotic except 1× MIC gentamicin, to avoid extracellular growth contamination and subsequent cell lysis (15).

### Accumulation and release experiments.

Gepotidacin accumulation and release were measured using a general protocol developed in our laboratory to study the cellular pharmacokinetics of antibiotics in J774 macrophages and THP-1 monocytes (13, 38). In brief, cells were incubated in the presence of gepotidacin (with a tracing amount of ^14^C-labeled drug) for the lengths of time and at the concentrations adapted for the purpose of each experiment. At the end of the incubation period, cells were washed with PBS and lysed by sonication, and the antibiotic concentration in the lysate was determined by scintillation counting and normalized by reference to the total cell protein content. The apparent cellular accumulation was then calculated using a conversion factor of 3.08 μl of cell volume per mg of cell protein for J774 macrophages (39) and 5 μl of cell volume per mg of cell protein for THP-1 monocytes (15).

### Cell fractionation studies.

We followed the general protocol developed in our laboratory for studying the subcellular distribution of antibiotics in cells (13, 18, 27, 40). In brief, uninfected cells were incubated with 1 mg/liter of gepotidacin (with a tracing amount of ^14^C-labeled drug) for 30 min, washed, and collected in ice-cold 0.25 M sucrose–3 mM Na EDTA–3 mM imidazole (pH 7.4) (sucrose-EDTA-imidazole), whereas infected cells were incubated with bacteria to allow phagocytosis as described above, washed, returned to fresh medium for 2 h to allow the complete internalization of bacteria, and then incubated for 30 min with gepotidacin at a total concentration of 0.1 mg/liter (to avoid killing of the bacteria), washed, and finally, collected in sucrose-EDTA-imidazole. Cells were then homogenized in the same medium using a Dounce tissue grinder. Subcellular organelles were separated by differential and isopycnic centrifugation as described previously (13, 35). In brief, for differential centrifugation, homogenates were separated into 3 successive fractions (nuclei and unbroken cells, organelles, and supernatant [cytosol]) by successive centrifugations (1,600, 25,000, and 40,000 rpm for 10 min, 6.7 min, and 30 min, respectively; the first one was conducted in a Beckman Allegra X-12R benchtop centrifuge and the last two were conducted in a rotor Ti50 operated in a Beckman Optima LE-80K ultracentrifuge [Beckman Coulter Life Sciences, Indianapolis, IN]). For isopycnic centrifugation, the cell homogenate was first made free of nuclei and unbroken cells by centrifugation at 1,600 rpm for 10 min, and the resulting cytoplasmic extract was deposited on top of a linear sucrose gradient with densities spanning from 1.10 to 1.24 resting on a cushion of sucrose of 1.34 density. After centrifugation at 39,000 rpm for 3 h in a swing-out rotor (SWTi50; Beckman), the gradient was collected into 12 discrete fractions, the densities of which were measured by refractometry (ABBE-3L refractometer; Bausch and Lomb, Rochester, NY). All fractions were assayed for protein content, radioactivity, the activity of marker enzymes (35), and, if infected, viable bacteria (counting of the number of CFU).

### Curve fitting and statistical analyses.

Curve fitting and statistical analyses were performed with GraphPad Prism (versions 4.03 and 7.03) and GraphPad InStat (version 3.10) software (GraphPad Software Inc., San Diego, CA) and JMP Pro (version 12.0.1) software (SAS Institute Inc., Cary, NC).

## References

[1] World Health Organization. WHO publishes list of bacteria for which new antibiotics are urgently needed. World Health Organization, Geneva, Switzerland http://www.who.int/mediacentre/news/releases/2017/bacteria-antibiotics-needed/en/ Accessed 10 August 2017.

[2] Clement S, Vaudaux P, Francois P, Schrenzel J, Huggler E, Kampf S, Chaponnier C, Lew D, Lacroix JS 2005 Evidence of an intracellular reservoir in the nasal mucosa of patients with recurrent Staphylococcus aureus rhinosinusitis. J Infect Dis 192:1023–1028. doi:10.1086/432735. 16107955

[3] Bosse MJ, Gruber HE, Ramp WK 2005 Internalization of bacteria by osteoblasts in a patient with recurrent, long-term osteomyelitis. A case report. J Bone Joint Surg Am 87:1343–1347. doi:10.2106/00004623-200506000-00022. 15930546

[4] Zautner AE, Krause M, Stropahl G, Holtfreter S, Frickmann H, Maletzki C, Kreikemeyer B, Pau HW, Podbielski A 2010 Intracellular persisting Staphylococcus aureus is the major pathogen in recurrent tonsillitis. PLoS One 5:e9452. doi:10.1371/journal.pone.0009452. 20209109 PMC2830486

[5] Van Bambeke F, Barcia-Macay M, Lemaire S, Tulkens PM 2006 Cellular pharmacodynamics and pharmacokinetics of antibiotics: current views and perspectives. Curr Opin Drug Discov Devel 9:218–230. 16566292

[6] Singh SB, Kaelin DE, Wu J, Miesel L, Tan CM, Meinke PT, Olsen D, Lagrutta A, Bradley P, Lu J, Patel S, Rickert KW, Smith RF, Soisson S, Wei C, Fukuda H, Kishii R, Takei M, Fukuda Y 2014 Oxabicyclooctane-linked novel bacterial topoisomerase inhibitors as broad spectrum antibacterial agents. ACS Med Chem Lett 5:609–614. doi:10.1021/ml500069w. 24900889 PMC4027601

[7] Bax BD, Chan PF, Eggleston DS, Fosberry A, Gentry DR, Gorrec F, Giordano I, Hann MM, Hennessy A, Hibbs M, Huang J, Jones E, Jones J, Brown KK, Lewis CJ, May EW, Saunders MR, Singh O, Spitzfaden CE, Shen C, Shillings A, Theobald AJ, Wohlkonig A, Pearson ND, Gwynn MN 2010 Type IIA topoisomerase inhibition by a new class of antibacterial agents. Nature 466:935–940. doi:10.1038/nature09197. 20686482

[8] Smart DJ, Lynch AM 2012 Evaluating the genotoxicity of topoisomerase-targeted antibiotics. Mutagenesis 27:359–365. doi:10.1093/mutage/ger089. 22155972 PMC3331794

[9] Flamm RK, Farrell DJ, Rhomberg PR, Scangarella-Oman NE, Sader HS 2017 Gepotidacin (GSK2140944) in vitro activity against Gram-positive and Gram-negative bacteria. Antimicrob Agents Chemother 61:e00468-17. doi:10.1128/AAC.00468-17. 28483959 PMC5487655

[10] Biedenbach DJ, Bouchillon SK, Hackel M, Miller LA, Scangarella-Oman NE, Jakielaszek C, Sahm DF 2016 In vitro activity of gepotidacin, a novel triazaacenaphthylene bacterial topoisomerase inhibitor, against a broad spectrum of bacterial pathogens. Antimicrob Agents Chemother 60:1918–1923. doi:10.1128/AAC.02820-15. 26729499 PMC4776004

[11] Farrell DJ, Sader HS, Rhomberg PR, Scangarella-Oman NE, Flamm RK 2017 In vitro activity of gepotidacin (GSK2140944) against Neisseria gonorrhoeae. Antimicrob Agents Chemother 61:e02047-16. doi:10.1128/AAC.02047-16. 28069643 PMC5328517

[12] O'Riordan W, Tiffany C, Scangarella-Oman N, Perry C, Hossain M, Ashton T, Dumont E 2017 Efficacy, safety, and tolerability of gepotidacin (GSK2140944) in the treatment of patients with suspected or confirmed Gram-Positive acute bacterial skin and skin structure infections. Antimicrob Agents Chemother 61:e02095-16. doi:10.1128/AAC.02095-16. 28373199 PMC5444153

[13] Peyrusson F, Butler D, Tulkens PM, Van Bambeke F 2015 Cellular pharmacokinetics and intracellular activity of the novel peptide deformylase inhibitor GSK1322322 against Staphylococcus aureus laboratory and clinical strains with various resistance phenotypes: studies with human THP-1 monocytes and J774 murine macrophages. Antimicrob Agents Chemother 59:5747–5760. doi:10.1128/AAC.00827-15. 26169402 PMC4538490

[14] Yoshida H, Bogaki M, Nakamura S, Ubukata K, Konno M 1990 Nucleotide sequence and characterization of the Staphylococcus aureus norA gene, which confers resistance to quinolones. J Bacteriol 172:6942–6949. doi:10.1128/jb.172.12.6942-6949.1990. 2174864 PMC210814

[15] Barcia-Macay M, Lemaire S, Mingeot-Leclercq MP, Tulkens PM, Van Bambeke F 2006 Evaluation of the extracellular and intracellular activities (human THP-1 macrophages) of telavancin versus vancomycin against methicillin-susceptible, methicillin-resistant, vancomycin-intermediate and vancomycin-resistant Staphylococcus aureus. J Antimicrob Chemother 58:1177–1184. doi:10.1093/jac/dkl424. 17062609

[16] Lemaire S, Glupczynski Y, Duval V, Joris B, Tulkens PM, Van Bambeke F 2009 Activities of ceftobiprole and other cephalosporins against extracellular and intracellular (THP-1 macrophages and keratinocytes) forms of methicillin-susceptible and methicillin-resistant Staphylococcus aureus. Antimicrob Agents Chemother 53:2289–2297. doi:10.1128/AAC.01135-08. 19289525 PMC2687181

[17] Buyck JM, Lemaire S, Seral C, Anantharajah A, Peyrusson F, Tulkens PM, Van Bambeke F 2016 In vitro models for the study of the intracellular activity of antibiotics. Methods Mol Biol 1333:147–157. doi:10.1007/978-1-4939-2854-5_13. 26468107

[18] Van Bambeke F, Carryn S, Seral C, Chanteux H, Tyteca D, Mingeot-Leclercq MP, Tulkens PM 2004 Cellular pharmacokinetics and pharmacodynamics of the glycopeptide antibiotic oritavancin (LY333328) in a model of J774 mouse macrophages. Antimicrob Agents Chemother 48:2853–2860. doi:10.1128/AAC.48.8.2853-2860.2004. 15273091 PMC478544

[19] Lemaire S, Tulkens PM, Van Bambeke F 2011 Contrasting effects of acidic pH on the extracellular and intracellular activities of the anti-Gram-positive fluoroquinolones moxifloxacin and delafloxacin against Staphylococcus aureus. Antimicrob Agents Chemother 55:649–658. doi:10.1128/AAC.01201-10. 21135179 PMC3028753

[20] Hamza T, Dietz M, Pham D, Clovis N, Danley S, Li B 2013 Intra-cellular Staphylococcus aureus alone causes infection in vivo. Eur Cell Mater 25:341–350. doi:10.22203/eCM.v025a24. 23832687 PMC3830899

[21] Ou J, Drilling A, Singhal D, Tan NC, Wallis-Hill D, Vreugde S, Psaltis AJ, Wormald PJ 2016 Association of intracellular Staphylococcus aureus with prognosis in chronic rhinosinusitis. Int Forum Allergy Rhinol 6:792–799. doi:10.1002/alr.21758. 27080195

[22] Tan NC, Foreman A, Jardeleza C, Douglas R, Vreugde S, Wormald PJ 2013 Intracellular Staphylococcus aureus: the Trojan horse of recalcitrant chronic rhinosinusitis? Int Forum Allergy Rhinol 3:261–266. doi:10.1002/alr.21154. 23423994

[23] Vallet CM, Marquez B, Ngabirano E, Lemaire S, Mingeot-Leclercq MP, Tulkens PM, Van Bambeke F 2011 Cellular accumulation of fluoroquinolones is not predictive of their intracellular activity: studies with gemifloxacin, moxifloxacin and ciprofloxacin in a pharmacokinetic/pharmacodynamic model of uninfected and infected macrophages. Int J Antimicrob Agents 38:249–256. doi:10.1016/j.ijantimicag.2011.05.011. 21764262

[24] Carlier MB, Garcia-Luque I, Montenez JP, Tulkens PM, Piret J 1994 Accumulation, release and subcellular localization of azithromycin in phagocytic and non-phagocytic cells in culture. Int J Tissue React 16:211–220. 7558665

[25] Melard A, Garcia LG, Das D, Rozenberg R, Tulkens PM, Van Bambeke F, Lemaire S 2013 Activity of ceftaroline against extracellular (broth) and intracellular (THP-1 monocytes) forms of methicillin-resistant Staphylococcus aureus: comparison with vancomycin, linezolid and daptomycin. J Antimicrob Chemother 68:648–658. doi:10.1093/jac/dks442. 23188792

[26] de Duve C, de Barsy T, Poole B, Trouet A, Tulkens P, Van Hoof F 1974 Commentary. Lysosomotropic agents. Biochem Pharmacol 23:2495–2531. doi:10.1016/0006-2952(74)90174-9. 4606365

[27] Lemaire S, Tulkens PM, Van Bambeke F 2010 Cellular pharmacokinetics of the novel biaryloxazolidinone radezolid in phagocytic cells: studies with macrophages and polymorphonuclear neutrophils. Antimicrob Agents Chemother 54:2540–2548. doi:10.1128/AAC.01723-09. 20385873 PMC2876419

[28] Lopez de Armentia MM, Amaya C, Colombo MI 2016 Rab GTPases and the autophagy pathway: bacterial targets for a suitable biogenesis and trafficking of their own vacuoles. Cells 5:E11. doi:10.3390/cells5010011. 27005665 PMC4810096

[29] Flannagan RS, Heit B, Heinrichs DE 2016 Intracellular replication of Staphylococcus aureus in mature phagolysosomes in macrophages precedes host cell death, and bacterial escape and dissemination. Cell Microbiol 18:514–535. doi:10.1111/cmi.12527. 26408990

[30] So W, Crandon JL, Nicolau DP 2015 Pharmacodynamic profile of GSK2140944 against methicillin-resistant Staphylococcus aureus in a murine lung infection model. Antimicrob Agents Chemother 59:4956–4961. doi:10.1128/AAC.00625-15. 26055376 PMC4505212

[31] von Eiff C, Becker K, Metze D, Lubritz G, Hockmann J, Schwarz T, Peters G 2001 Intracellular persistence of Staphylococcus aureus small-colony variants within keratinocytes: a cause for antibiotic treatment failure in a patient with Darier's disease. Clin Infect Dis 32:1643–1647. doi:10.1086/320519. 11340539

[32] Stepinska M, Olszewska-Sosinska O, Lau-Dworak M, Zielnik-Jurkiewicz B, Trafny EA 2014 Identification of intracellular bacteria in adenoid and tonsil tissue specimens: the efficiency of culture versus fluorescent in situ hybridization (FISH). Curr Microbiol 68:21–29. doi:10.1007/s00284-013-0436-0. 23934353

[33] Tsuchiya S, Yamabe M, Yamaguchi Y, Kobayashi Y, Konno T, Tada K 1980 Establishment and characterization of a human acute monocytic leukemia cell line (THP-1). Int J Cancer 26:171–176. doi:10.1002/ijc.2910260208. 6970727

[34] Snyderman R, Pike MC, Fischer DG, Koren HS 1977 Biologic and biochemical activities of continuous macrophage cell lines P388D1 and J774.1. J Immunol 119:2060–2066. 915291

[35] Renard C, Vanderhaeghe HJ, Claes PJ, Zenebergh A, Tulkens PM 1987 Influence of conversion of penicillin G into a basic derivative on its accumulation and subcellular localization in cultured macrophages. Antimicrob Agents Chemother 31:410–416. doi:10.1128/AAC.31.3.410. 3579258 PMC174742

[36] Scorneaux B, Ouadrhiri Y, Anzalone G, Tulkens PM 1996 Effect of recombinant human gamma interferon on intracellular activities of antibiotics against Listeria monocytogenes in the human macrophage cell line THP-1. Antimicrob Agents Chemother 40:1225–1230. 8723471 10.1128/aac.40.5.1225 PMC163296

[37] Clinical and Laboratory Standards Institute. 2017 Performance standards for antimicrobial susceptibility testing; 24th informational supplement (MS100-S27). Clinical and Laboratory Standards Institute, Wayne, PA.

[38] Lemaire S, Van Bambeke F, Appelbaum PC, Tulkens PM 2009 Cellular pharmacokinetics and intracellular activity of torezolid (TR-700): studies with human macrophage (THP-1) and endothelial (HUVEC) cell lines. J Antimicrob Chemother 64:1035–1043. doi:10.1093/jac/dkp267. 19759040

[39] Michot JM, Van Bambeke F, Mingeot-Leclercq MP, Tulkens PM 2004 Active efflux of ciprofloxacin from J774 macrophages through an MRP-like transporter. Antimicrob Agents Chemother 48:2673–2682. doi:10.1128/AAC.48.7.2673-2682.2004. 15215125 PMC434197

[40] Carlier MB, Scorneaux B, Zenebergh A, Desnottes JF, Tulkens PM 1990 Cellular uptake, localization and activity of fluoroquinolones in uninfected and infected macrophages. J Antimicrob Chemother 26(Suppl B):27–39. 2258352 10.1093/jac/26.suppl_b.27

[41] Kosowska-Shick K, Clark C, Credito K, McGhee P, Dewasse B, Bogdanovich T, Appelbaum PC 2006 Single- and multistep resistance selection studies on the activity of retapamulin compared to other agents against Staphylococcus aureus and Streptococcus pyogenes. Antimicrob Agents Chemother 50:765–769. doi:10.1128/AAC.50.2.765-769.2006. 16436741 PMC1366917

[42] Kosowska-Shick K, Ednie LM, McGhee P, Smith K, Todd CD, Wehler A, Appelbaum PC 2008 Incidence and characteristics of vancomycin nonsusceptible strains of methicillin-resistant Staphylococcus aureus at Hershey Medical Center. Antimicrob Agents Chemother 52:4510–4513. doi:10.1128/AAC.01073-08. 18838583 PMC2592881

[43] Tsiodras S, Gold HS, Sakoulas G, Eliopoulos GM, Wennersten C, Venkataraman L, Moellering RC, Ferraro MJ 2001 Linezolid resistance in a clinical isolate of Staphylococcus aureus. Lancet 358:207–208. doi:10.1016/S0140-6736(01)05410-1. 11476839

[44] Kuroda M, Ohta T, Uchiyama I, Baba T, Yuzawa H, Kobayashi I, Cui L, Oguchi A, Aoki K, Nagai Y, Lian J, Ito T, Kanamori M, Matsumaru H, Maruyama A, Murakami H, Hosoyama A, Mizutani-Ui Y, Takahashi NK, Sawano T, Inoue R, Kaito C, Sekimizu K, Hirakawa H, Kuhara S, Goto S, Yabuzaki J, Kanehisa M, Yamashita A, Oshima K, Furuya K, Yoshino C, Shiba T, Hattori M, Ogasawara N, Hayashi H, Hiramatsu K 2001 Whole genome sequencing of meticillin-resistant Staphylococcus aureus. Lancet 357:1225–1240. doi:10.1016/S0140-6736(00)04403-2. 11418146

[45] Ba BB, Arpin C, Vidaillac C, Chausse A, Saux MC, Quentin C 2006 Activity of gatifloxacin in an in vitro pharmacokinetic-pharmacodynamic model against Staphylococcus aureus strains either susceptible to ciprofloxacin or exhibiting various levels and mechanisms of ciprofloxacin resistance. Antimicrob Agents Chemother 50:1931–1936. doi:10.1128/AAC.01586-05. 16723548 PMC1479150

